# Comparative FISH-Mapping of *MC1R*, *ASIP*, and *TYRP1* in New and Old World Camelids and Association Analysis With Coat Color Phenotypes in the Dromedary (*Camelus dromedarius*)

**DOI:** 10.3389/fgene.2019.00340

**Published:** 2019-04-16

**Authors:** Fahad Alshanbari, Caitlin Castaneda, Rytis Juras, Andrew Hillhouse, Mayra N. Mendoza, Gustavo A. Gutiérrez, Federico Abel Ponce de León, Terje Raudsepp

**Affiliations:** ^1^Department of Veterinary Integrative Biosciences, College of Veterinary Medicine and Biomedical Sciences, Texas A&M University, College Station, TX, United States; ^2^Institute for Genome Sciences and Society, Texas A&M University, College Station, TX, United States; ^3^Animal Breeding Program, National Agrarian University La Molina, Lima, Peru; ^4^Department of Animal Science, University of Minnesota, Minneapolis, MN, United States

**Keywords:** camelids, ASIP, MC1R, TYRP1, FISH, TaqMan assay

## Abstract

Melanocortin 1 receptor (*MC1R*), the agouti signaling protein (*ASIP*), and tyrosinase related protein 1 (*TYRP1*) are among the major regulators of pigmentation in mammals. Recently, *MC1R* and *ASIP* sequence variants were associated with white and black/dark brown coat colors, respectively, in the dromedary. Here we confirmed this association by independent sequencing and mutation discovery of *MC1R* and *ASIP* coding regions and by TaqMan genotyping in 188 dromedaries from Saudi Arabia and United States, including 38 black, 53 white, and 97 beige/brown/red animals. We showed that heterozygosity for a missense mutation c.901C > T in *MC1R* is sufficient for the white coat color suggesting a possible dominant negative effect. Likewise, we confirmed that the majority of black dromedaries were homozygous for a frameshift mutation in *ASIP* exon 2, except for 4 animals, which were heterozygous. In search for additional mutations underlying the black color, we identified another frameshift mutation in *ASIP* exon 4 and 6 new variants in *MC1R* including a significantly associated SNP in 3′UTR. In pursuit of sequence variants that may modify dromedary wild-type color from dark-reddish brown to light beige, we identified 4 SNPs and one insertion in *TYRP1* non-coding regions. However, none of these were associated with variations in wild-type colors. Finally, the three genes were cytogenetically mapped in New World (alpaca) and Old World (dromedary and Bactrian camel) camelids. The *MC1R* was assigned to chr21, *ASIP* to chr19 and *TYRP1* to chr4 in all 3 species confirming extensive conservation of camelid karyotypes. Notably, while the locations of *ASIP* and *TYRP1* were in agreement with human-camelid comparative map, mapping *MC1R* identified a new evolutionary conserved synteny segment between camelid chromosome 21 and HSA16. The findings contribute to coat color genomics and the development of molecular tests in camelids and toward the chromosome level reference assemblies of camelid genomes.

## Introduction

Mammalian coat color is a phenotypic trait that serves for camouflage and communication in the wild, and has been a target for selection by humans in farm and companion species since their domestication (Andersson, 2001; Cieslak et al., 2011). As a result, domestic animals display a perplexing variety of colors, patterns and markings, which reflect the genetic diversity of a breed or species, as well as historic and aesthetic preferences or commercial needs of humans.

Many genes regulate coat color. This was already noted by Haldane (1927) over 90 years ago when he studied color genetics in rodents and carnivores and suggested that there are at least 20 different color genes in mammals. Since then, approximately 150 coat-color associated genes have been described in mice, humans, and domestic animals (Schmutz and Berryere, 2007; Bellone, 2010; Cieslak et al., 2011; Reissmann and Ludwig, 2013), whereas *Color Genes* database^1^ lists 378 mouse loci with their human and zebrafish homologs that are associated with various pigmentation phenotypes.

Despite the large number of genes involved, the production, amount and distribution of main pigments, the brown/black eumelanin and the red/yellow pheomelanin, are controlled by just a few major pigmentation genes (Rees, 2003; Bellone, 2010; Reissmann and Ludwig, 2013; Suzuki, 2013). These include melanocortin 1 receptor (*MC1R*), agouti signaling protein (*ASIP*) and tyrosinase related protein 1 (*TYRP1*). Melanocortin 1 receptor is the key switch between the synthesis of eumelanin or pheomelanin; ASIP is an antagonist ligand that regulates MC1R signaling by inhibiting the MC1R receptor, and TYRP1 is a melanogenic enzyme that influences the quantity and quality of melanins (Pielberg, 2004; Bellone, 2010; Sturm and Duffy, 2012; Suzuki, 2013). Associations between basic coat colors and DNA sequence polymorphisms in *MC1R*, *ASIP*, *TYRP1*, are known for most domestic species (Rieder et al., 2001; Pielberg, 2004; Schmutz and Berryere, 2007; Bellone, 2010; Cieslak et al., 2011), and are routinely used for genetic testing.

In contrast to other domestic species, coat color genomics in camelids had a late start, even though fiber color is an important trait for the alpaca industry (Morante et al., 2009) and there is an interest for breeding white or black dromedaries in some Arabian countries (Almathen et al., 2018). A few studies in alpacas have associated mutations in *ASIP* with the black color (Feeley et al., 2011; Chandramohan et al., 2013) and identified *MC1R* mutations that may determine light phenotypes, though the findings about the alpaca *MC1R* remain inconclusive (Feeley and Munyard, 2009; Guridi et al., 2011; Chandramohan et al., 2015). Research on dromedary color genes is even more recent with just two publications. The first study revealed that a frameshift mutation in the *KIT* gene explains some, though not all forms of white-spotting phenotypes in the dromedary (Holl et al., 2017). The most recent study identified a missense mutation in *MC1R* that is associated with the white color, and a deletion and a single nucleotide polymorphism (SNP) in *ASIP* exon 2 that are associated with the black/dark brown color in dromedaries (Almathen et al., 2018). Current reference genomes for the alpaca and the dromedary are in scaffolds and not assigned to chromosomes^2^. Because of this, chromosomal location is known only for the few coat color genes that were included in the alpaca whole genome cytogenetic map (Avila et al., 2014a). Among the main pigmentation genes, *ASIP* and *TYRP1* have been mapped in the alpaca but not in other camelids, whereas *MC1R* is not mapped in any camelid species.

The aim of this study is to confirm and refine the recently reported *MC1R* and *ASIP* mutations for white and black coat color in dromedaries, and search for novel color-related variants in *TYRP1*. We compare the accuracy of genotyping the white and black mutations in large dromedary populations by direct sequencing and with a TaqMan^TM^ assay. Finally, we cytogenetically map *ASIP*, *MC1R*, and *TYRP1* in three camelid species.

## Materials and Methods

### Ethics Statement

Procurement of peripheral blood was performed according to the United States Government Principles for the Utilization and Care of Vertebrate Animals Used in Testing, Research and Training. These protocols were approved by Animal Use Protocol AUP #2011-96, # 2018-0342 CA and CRRC #09-47 at Texas A&M University.

### Animals and Phenotypes

**Figure 1:**
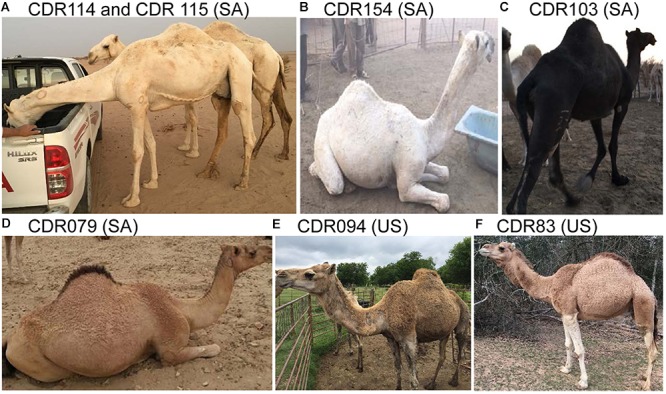
Examples of animals and coat colors used for this study. **(A)** White/cream; **(B)** White; **(C)** Black; **(D)** Reddish brown with dark hump and tail; **(E)** Medium brown; and **(F)** Light brown; SA, Saudi Arabia; US, United States; dromedary IDs and genotypes are in Supplementary Table S1.

We sampled 188 dromedaries originating from Saudi Arabia (SA; *n* = 171) and from the United States (US; *n* = 17). Coat color phenotypes were determined by visual inspection, recorded in written notes and/or photos, and were as follows: white/cream (*n* = 53), black/dark brown (*n* = 38), and brown/beige (*n* = 97) (Figure 1). Two brown dromedaries had white markings and blue eyes. We use ‘*brown*’ as a generic term to denote animals with wild-type coat color, which can range from light beige to darker reddish–brown with either matching or darker tail and hump. Supplementary Table S1 presents summary information for all animals and phenotypes.

### Samples

Blood was collected by jugular venipuncture into EDTA-containing Vacutainers (Becton Dickinson).

### DNA Isolation

Genomic DNA was isolated from peripheral blood lymphocytes using Gentra Puregene Blood Kit (Qiagen) following the manufacturer’s protocol, or by standard phenol-chloroform method (Sambrook et al., 1989). We evaluated DNA quality and quantity by NanoDrop 2000 spectrophotometer (Thermo Scientific) and by 1% agarose gel electrophoresis.

### Primers, PCR and Sequencing

We used the available sequence information for the dromedary and alpaca *MC1R*, *ASIP*, and *TYRP1* in NCBI^3^, UCSC^4^, and Ensembl^5^ genome browsers, or sequences of the Bactrian camel (Wu et al., 2014) and Primer3 software (Untergasser et al., 2012) to design primers. For *ASIP* and *TYRP1*, primers were designed to amplify all exons and exon–intron boundaries. For *MC1R*, primers were designed to amplify overlapping fragments covering the single exon and the 5′UTR. Primer details are presented in Table 1. PCR was conducted in 10 μL reactions containing 50 ng dromedary genomic DNA and 0.5 unit of JumpStart Taq ReadyMix (Sigma Aldrich). For *MC1R*, primers 5′UTR.1 F and 5′UTR.2 R (Table 1) were combined to amplify the entire 2 kb of the 5′UTR. The PCR products were cleaned using ExoSAP (Affymetrix) and sequenced using BigDye Terminator v1.1 Cycle Sequencing Kit (Applied Biosystems) and the manufacturer’s protocol. Sequencing reactions were cleaned in Spin-50 mini columns (BioMax, Inc) and resolved on 3100 automated sequencer (Applied Biosystems).

**Table 1:** Primers used for PCR and sequencing of *MC1R*, *ASIP*, and *TYRP1.*

Gene symbol	Region	Forward 5′ – 3′	Reverse 5′ – 3′	Product
and primer ID				size, bp
*MC1R* 5′ UTR 1	5′ UTR	TCTCACGCCCTTTGAAGTCT	GCTGACGACAAACCCTTCTC	2007
*MC1R* 5′ UTR 2	5′ UTR	ACACACCTTAACGGGACACC	GCAGGAAAGGGTCTTCACTCT	1101
*MC1R* 1	Exon 1	TCCTCCTCTGTCTCGTCAGC	GTTGGCTGGCACTGTCTCC	918
*MC1R* 2	Exon 1	GGCGCTGTCTCTTGTGGAG	GACCAGAAGAGACGCAGGAG	912
*MC1R* 3	Exon 1	CTCCCTGGCAGGACGATG	AGTCCGAGGTGGGTGGTG	92
*MC1R*_OV**^∗^**	Exon 1	CATGGTGTCCAGCCTCTGCTCTCT	CACGGCGATAGCACCCAGAGAGCA	n/a
*ASIP* Prom	Promoter	GGATTTGGGGTCAGTCTGTA	CCCATCCCTTTAGCCTCCTA	2608
*ASIP* Ex1	Exon 1	GTGTGAGTCAGTGGCAGGAA	AAATTCTGGGTGGGCTAAGG	1130
*ASIP* Ex1b	Exon 1	ACTTAAGGCAGGCTGGACCT	ATGTGCCCATCCCTTTAGC	1502
*ASIP*ex1seq	Exon 1	AGACCCTGCATTAAGCTGCTC	Sequencing primer	n/a
*ASIP*ex1seqb	Exon 1	GCTTTTCTGATAATGAAATA	Sequencing primer	n/a
*ASIP* Ex 2	Exon 2	CTTCAGTCTCCCTCCCTTCC	GCCAGGTATTTTTCCCTGAG	828
*ASIP* Ex 3	Exon 3	TCCAGGGCCTTATTGGACTT	CTGGAAAGGCTCAGTTTGCT	953
*ASIP* Ex 4	Exon 4	ACTGTAAGAGGGCCAGAGCA	TAAAGTAGGGGGCAGCATTG	511
*TYRP1* Ex 1	Exon 1	AGCACTTTGAAGGTGGGTTG	AGTCAGAAGACTGGAGCATCAA	698
*TYRP1* Ex 2	Exon 2	AAGAGAGGGAGTGGAAGGGAGA	ATGTGAAATTGCTTGGTCAGTG	650
*TYRP1* Ex 3	Exon 3	TGAGTTGGGTTTCATTCCTT	CACTTTCTTTTTCCCCTGGA	629
*TYRP1* Ex 4	Exon 4	TGGACATGGTAACTTGGGTTT	GGCCAGCAACCTAACTTTGA	722
*TYRP1* Ex 5	Exon 5	GGCCACCAACCATAGGTACA	GACTTCCTGTCTGCCTTTTCA	525
*TYRP1* Ex 6	Exon 6	CCTGGGCTGCTGTAGTGAA	CTGGGGGCTCTCAACAAACT	500
*TYRP1* Ex 7	Exon 7	GGAATTAGGAAGTGCCCTGA	AACATGCCCCAAATCTTCAC	595

*Asterisk denotes overgo primers for screening *MC1R* from CHORI-246 BAC library*.

### Sequence Analysis and Mutation Discovery

For initial mutation discovery, we sequenced PCR products of *MC1R*, *ASIP*, and *TYRP1* in 4 white, 4 black, and 4 brown dromedaries. Sequences were analyzed for mutations using Sequencher v 5.3 software (Gene Codes Corp.). Effects of single nucleotide changes and indels on protein structure and function were evaluated with Protein Variation Effect Analyzer (PROVEAN) toolkit^6^ (Choi et al., 2012; Choi and Chan, 2015). Amino acid sequences of different species were retrieved from NCBI^7^ and Ensembl^8^. Comparative analysis of the MC1R protein across species was performed by aligning amino acid sequences in ClustalW (Thompson et al., 1994). We used Transmembrane Protein Topology with a Hidden Markov Model^9^ (Moller et al., 2001) to determine MC1R transmembrane domains and evaluate the effect of SNPs; GeneCluster 2.0 (Reich et al., 2004) for comparative analysis of MC1R across species, and ExPASy webtools (Gasteiger et al., 2003) to translate genomic sequence into protein.

### Large Cohort Genotyping and Association Analysis

Putative causative mutations in *MC1R* and *ASIP* were further analyzed for genotype–phenotype association by Sanger sequencing the regions in 69 dromedaries (29 white, 17 black, 23 brown). Custom TaqMan^TM^ SNP genotyping assays were designed for *MC1R* and *ASIP* mutations according to manufacturer specification (Applied Biosystems) (Table 2), and used for genotyping all 188 dromedaries. We used CFX-96 Real Time-PCR machine (Bio-Rad) and corresponding software for PCR amplifications, genotyping and allelic discrimination. The thermal conditions were: priming at 60°C for 1 min, initial denaturation at 95°C for 10 min, 40 cycles of 92°C for 15 s, annealing at primer-specific t°C, extension for 1 min at 60°C, followed by a final extension at 65°C. The 8 μL reactions contained 0.208 μL of TaqMan^TM^ assay, 30 ng template DNA and 4.2 μL of ABI TaqMan Universal Master mix, no UNG (Applied Biosystems).

**Table 2:** TaqMan assays for genotyping *MC1R* g901C > T and *ASIP* g.174495T > Del.

Primer/probe	5′-3′
*MC1R*-forward	CTCATCATCTGCAACTCCATCGT
*MC1R*-reverse	CAGCACCTCTTGGAGTGTCTTC
*MC1R*_VICprobe	ATGCCTTCCGCAGCCA
*MC1R*_FAMprobe	CTATGCCTTCTGCAGCCA
*ASIP*-forward	CCACTCAGATATCCCAGGATGGA
*ASIP*-reverse	GCTGTAGGCATTGAGGAAGCA
*ASIP*_VICprobe	CCTCTTCCTAGCTACCC
*ASIP*_FAMprobe	CCTCTTCCAACTACCC

### Statistical Analysis

We conducted contingency analysis with JMP program v12 (JMP^®^, Version 13. SAS Institute Inc., Cary, NC, United States, 1989–2007) to examine the relationship between color phenotypes and genotypes at each variable site. Contingency analysis explores the distribution of a categorical variable Y (color phenotypes) across the level of a second categorical variable X (genotypes). The analysis results include three output files: a mosaic plot, contingency table, and statistical tests (Supplementary Figure S2). The mosaic plot is divided into rectangles, so that the vertical length of each rectangle is proportional to the proportions of the observed phenotypes (the Y variable) in each genotype (the X variable). The contingency table is a two-way frequency table with a row for each genotype and a column for each phenotype, and shows their total count, total percent, phenotype percent and genotype percent relative to the total number of observations. The last part of the report shows the results of statistics tests to determine whether or not the phenotypes are independent from genotypes and include R-square and two Chi-square tests, and a probability estimation (Prob > ChiSq) (see Supplementary Figure S2 for more details).

### Chromosome Preparations

Alpaca, dromedary and Bactrian camel chromosome slides were prepared from methanol:acetic acid (3:1)-fixed cell suspensions available in the depository of the Molecular Cytogenetics laboratory at Texas A&M University. All cell suspensions originated from normal individuals with normal karyotypes.

### Fluorescence *in situ* Hybridization (FISH)

We used alpaca CHORI-246 genomic Bacterial Artificial Chromosome (BAC) library^10^ to obtain probes for FISH. BAC clones containing *ASIP* and *TYRP1* were previously identified and mapped in the alpaca (Avila et al., 2014a). To obtain BACs for *MC1R*, we screened CHORI-246 filters with *MC1R*-specific radioactively labeled ([^32^P] dATP/dCTP) overgo primers (Table 1) as described by Avila et al. (2014b). The final BACs containing *MC1R* were further verified by PCR with *MC1R* exon primers (Table 1). BAC DNA was isolated with Plasmid Mini Kit (Qiagen) according to the manufacturer’s protocol. Probe labeling, hybridization and signal detection were conducted according to standard protocols (Raudsepp and Chowdhary, 2008). Because of difficulties to unambiguously identify camelid chromosomes by conventional cytogenetic methods (Avila et al., 2014b), BACs containing the three genes were co-hybridized with a differently labeled reference gene from the alpaca cytogenetic map (Avila et al., 2014a). Composite information about the BACs used for comparative FISH mapping is presented in Table 3. Images for at least 10 metaphases for each experiment were captured and analyzed using a Zeiss Axioplan 2 fluorescence microscope, equipped with the Isis Version 5.2 (MetaSystems GmbH) software.

**Table 3:** Comparative cytogenetic mapping of *ASIP*, *MC1R*, and *TYRP1*.

CHORI-246 BAC	Gene symbol	Camelid chr.	Alpaca	Dromedary	Bactrian	Human chr.
018C13	*ASIP*	19q12	Avila et al., 2014a	This study	This study	20q11.2-q12
125P19^∗^	*EDN3^∗^*	19q23	Avila et al., 2014a	This study	This study	20q13.2-q13.3
166N17	*MC1R*	21q15	This study	This study	This study	16q23
128F16^∗^	*MYOC^∗^*	21q13	Avila et al., 2014a	This study	This study	1q23-q24
129N17	*TYRP1*	4q21dist-q22	Avila et al., 2014a	This study	This study	9p23
135B22^∗^	*MRPL41^∗^*	4q36	Avila et al., 2014a	This study	This study	9q34.3

*Details about alpaca BAC clones, corresponding genes and cytogenetic locations; ^∗^denotes reference BACs/genes for chromosome identification*.

## Results

### Mutation Discovery and Association Analysis of *MC1R*

**Figure 2:**
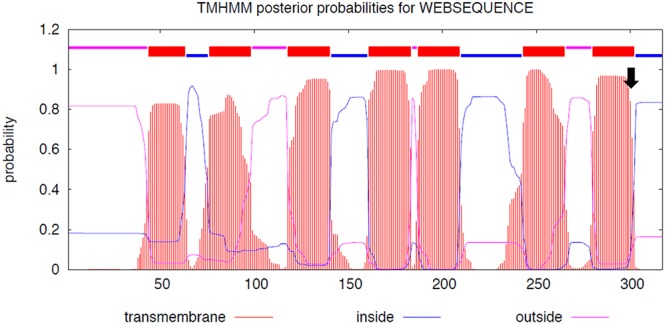
MC1R protein functional domains. The seven MC1R transmembrane domains and the position of p.301R > C mutation (arrow); *y*-axis: the probability of the amino acid sequences to be cytoplasmic (blue), extracellular (magenta), or part of the transmembrane helix (orange); *x*-axis: amino acid sequence. We used Transmembrane Protein Topology with a Hidden Markov Model (Moller et al., 2001; http://www.cbs.dtu.dk/services/TMHMM/TMHMM2.0b.guide.php).

The initial sequence analysis of 12 individuals (4 white, 4 black, and 4 brown) identified 7 sequence variants inside and around *MC1R* (Table 4). All variants were SNPs and included the previously reported c.901C > T (p.Arg301Cys) missense mutation in the *MC1R* coding region (Almathen et al., 2018) and 6 new non-coding variants: three SNPs in the promoter region, two in 5′UTR and one in 3′UTR. The c.901C > T missense mutation was genotyped in large cohorts by Sanger sequencing (*n* = 68) and by TaqMan^TM^ genotyping (*n* = 188) showing that this mutation is significantly associated (*P* < 0.0001) with the white color (Table 5), thus confirming the findings of Almathen et al. (2018). To evaluate the possible effect of the p.301R > C mutation on MC1R function, we constructed transmembrane protein topology and showed that the amino acid change affects the last of the 7 transmembrane domains (Figure 2). We also aligned the amino acid sequences of the MC1R last transmembrane domain in diverse mammalian and vertebrate species and showed that arginine at this position is highly conserved across species (Supplementary Figure S1), suggesting its importance for MC1R normal function.

**Table 4:** Sequence polymorphisms in *ASIP*, *MC1R*, and *TYRP1.*

Gene symbol	Location in the gene	Variant	Reference	Effect on protein	Phenotype–genotype			*P-value*
					White	Black	Brown	
*MC1R*	Promoter (1802 bp from ORF	g.535236C > T	This study	Non-coding	4CC	4CT	4CT	0.0005
*MC1R*	Promoter (557 bp from ORF)	g.536482G > A	This study	Non-coding	2GG, 2GA	1GG, 2GA, 1AA	2GG, 2GA	0.6208
*MC1R*	Promoter (420 bp from ORF)	g.536623G > A	This study	Non-coding	4GG	3GG, 1GA	3GG, 1GA	0.4033
*MC1R*	5′UTR	g.537027G > A	This study	Non-coding	4GG	3GG, 1GA	3GG, 1GA	0.4033
*MC1R*	5′UTR	g.537028A > T	This study	Non-coding	4AA	3AA, 1AT	3AA, 1AT	0.4033
*MC1R*	Exon	g.537961C > T; c.901C > T	Almathen et al., 2018	Missense; p.Arg301Cys	3CT, 1TT	4CC	4CC	0.0042
*MC1R*	3′UTR	g.538058G > A	This study	Non-coding	4GG	2GG, 2GA	4GG	0.0718
*ASIP*	Exon 2	g.174495T_del; c.23T_del	Almathen et al., 2018	Frameshift; p.24X	4TD	4DD	2TT,2TD	0.0009
*ASIP*	Exon 2	g.174497A > G; c.25A > G	Almathen et al., 2018	Synonymous	4GA	4AA	2GG, 2GA	0.0009
*ASIP*	Exon 4	g.178388C_del	This study	Frameshift; p.253X	4CC	2CC, 2CD	3CC, 1CD	0.178
*TYRP1*	Intron 1 (72 bp before exon 2	g.6544484G > A	This study	Non-coding	4GG	3GG, 1GA	4GG	0.3359
*TYRP1*	Intron 2 (26 bp after exon 2)	g.6544049T > C	This study	Non-coding	2TT, 2TC	4TT	1TT, 3TC	0.0438
*TYRP1*	Intron 3 (13 bp before exon 4)	g.65372260_g.65372261insCA	This study	Non-coding	1insCA/insCA	None	None	0.3359
*TYRP1*	Intron 4 (90 bp after exon 4	g.6537183T > C	This study	Non-coding	2TT, 2TC	3TT, 1TC	1TT, 3TC	0.3512
*TYRP1*	3′UTR	g.6529345A > T	This study	Non-coding	3AA, 1AT	3AA, 1AT	3AA, 1AT	1

*Sequence variants were discovered by sequencing the three genes in 4 white, 4 black, and 4 brown dromedaries. Sequence positions correspond to dromedary whole genome assembly: GCA_000767585.1 PRJNA234474_Ca_dromedarius_V1.0.; *MC1R* scaffold ID: NW_011592664.1, *ASIP* scaffold ID: NW_011591043.1 and *TYRP1* scaffold ID: NW_011591511.1; Numbers in columns White, Black, Brown denote the number of animals with the corresponding genotype; ORF, open reading frame; D, deletion; *P-value* for genotype–phenotype association was determined by contingency analysis in JMP using Chi-square (see Supplementary Figure S2)*.

**Table 5:** Genotype frequencies of *MC1R* c.901C > T missense mutation in a large study cohort (*n* = 188).

Genotypes	Frequency (count) in		
	dromedary color groups		
	White	Black	Brown
	(*n* = 53)	(*n* = 38)	(*n* = 97)
CC	0.037 (7)	0.20 (38)	0.41 (78)
CT	0.13 (25)	0	0.085 (16)
TT	0.11 (21)	0	0.016 (3)

*The mutation is significantly (*P* < 0.0001) associated with white coat color*.

The initial analysis also indicated that the SNP g.538058G > A in *MC1R* 3′UTR may be associated with color phenotype because genotype GA was present only in black dromedaries (Table 4). Large cohort (*n* = 68) genotyping by sequencing confirmed this and showed that GA genotype was more frequent (*P* < 0.0004; Table 6) in black animals. Notably, we did not find dromedaries homozygous for the A-allele (AA) at this site, which is most likely due to the low (0.08) minor-allele (A) frequency, which predicts AA-genotype frequency in small (*n* = 12) cohort as 0.01 and in large (*n* = 68) cohort as 0.44 (Supplementary Table S2).

**Table 6:** Genotype frequencies of *MC1R* 3′UTR variant g.538058G > A in a large study cohort (*n* = 68).

Genotypes	Frequency (count) in		
	dromedary color groups		
	White	Black	Brown
	(*n* = 22)	(*n* = 15)	(*n* = 31)
GG	0.29 (20)	0.13 (9)	0.46 (31)
GA	0.029 (2)	0.088 (6)	0
AA	0	0	0

*The SNP is significantly (*P* < 0.0004) associated with black coat color*.

### Mutation Discovery and Association Analysis of *ASIP*

**Figure 3:**
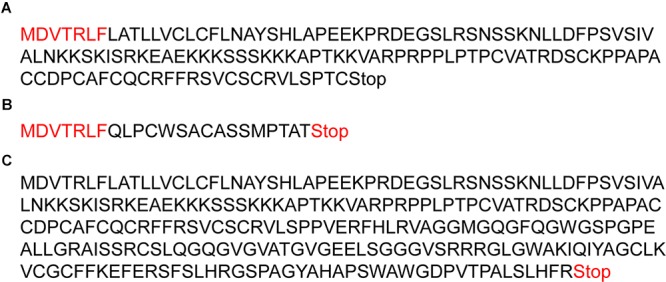
The effect of frameshift mutations on ASIP polypeptide. **(A)** Normal ASIP polypeptide with 133 amino acids and stop at codon 134; **(B)** Truncated ASIP protein with 24 amino acids and stop at codon 25 due to frameshift mutation in exon 2; **(C)** Abnormally long polypeptide with 253 amino acids due to a frameshift mutation in exon 4. Amino acids in red font in **(A,B)** are before frameshift and, thus shared between the normal and truncated ASIP.

We identified three sequence variants in the four exons of the dromedary *ASIP* gene: – two previously known in exon 2 (Almathen et al., 2018) and a new frameshift mutation exon 4 (Table 4). A single nucleotide deletion in exon 2 (g.174495T_del; c.23T_del) combined with a SNP two base-pairs later (g.174497A > G; c.25A > G; Table 4) caused a shift in the reading frame, an insertion of a premature stop at codon 24, and truncated protein (Figure 3). All black dromedaries in the discovery cohort were homozygous for the frameshift deletion. Therefore, we genotyped the frameshift mutation in large dromedary cohorts by sequencing (*n* = 68) and TaqMan^TM^ assay (*n* = 188). The results showed that the frameshift mutation in exon 2 is significantly associated (*P* < 0.0001) with black coat color (Table 7), consistent with the previous findings (Almathen et al., 2018). However, in the large study cohort, one black animal did not have the frameshift deletion and three black dromedaries were heterozygous for it (Table 7 and Supplementary Table S1). In these four animals, we analyzed *ASIP* sequence further and discovered that two dromedaries were heterozygous for another frameshift mutation in exon 4 at g.178388C_del (Table 4). The mutation shifted normal stop at codon 133 to codon 254, resulting in 120 amino acids longer polypeptide (Figure 3). However, the other two animals did not have this deletion and, overall, we were not able to associate exon 4 mutation with black color in our study cohort.

**Table 7:** Genotype frequencies of *ASIP* exon 2 g.174495T_del (D) nonsense mutation in a large study cohort (*n* = 188).

Genotypes	Frequency (count) in		
	dromedary color groups		
	White	Black	Brown
	(*n* = 53)	(*n* = 38)	(*n* = 97)
TT	0.12 (23)	0.005 (1)	0.20 (37)
TD	0.13 (25)	0.016 (3)	0.18 (33)
DD	0.03 (5)	0.18 (34)	0.14 (27)

*The mutation is significantly (*P* < 0.0001) associated with black coat color*.

### Mutation Discovery in *TYRP1*

Sequence analysis of the 7 exons and exon–intron boundaries of the *TYRP1* gene in the discovery cohort of 12 dromedaries, identified 5 sequence variants: 4 SNPs and one insertion (Table 4). However, all variants were in non-coding regions (introns and 3′UTR) and not associated with dromedary color phenotypes. Therefore, we did not conduct any large cohort genotyping for *TYRP1*.

### Comparative FISH Mapping

**Figure 4:**
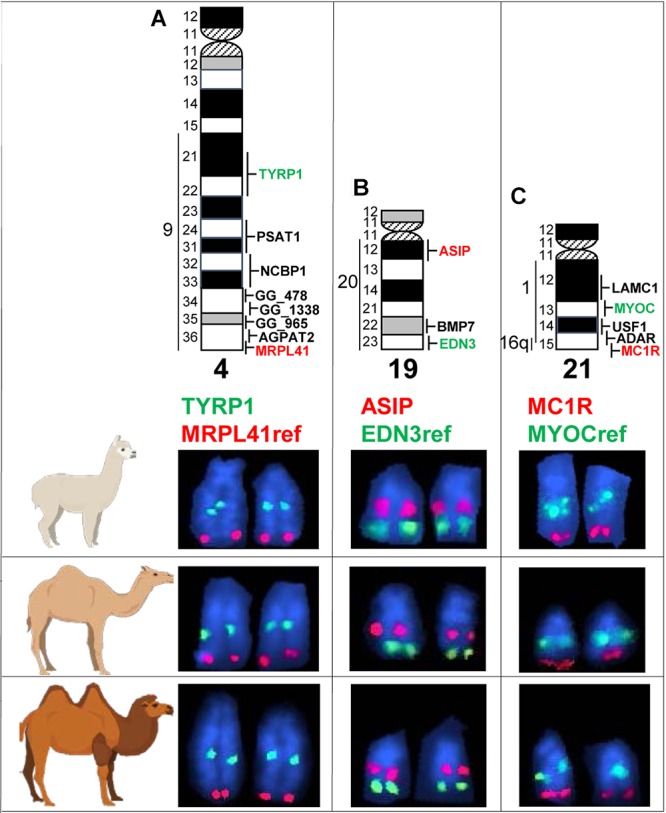
Comparative FISH mapping. Comparative mapping of *TYRP1* **(A)**, *ASIP* **(B)**, and *MC1R* **(C)** in alpaca, dromedary, and Bactrian camel chr4, chr19, and chr21, respectively. Chromosome ideograms with all mapped markers (Avila et al., 2014a) are shown at the top. Vertical lines with numbers to the left of chromosome ideograms indicate homology segments to human chromosomes. Ref, reference gene for chromosome identification. Green and red font colors for coat color genes and reference genes correspond to the green and red FISH signals in partial microscope images below ideograms.

We mapped *TYRP1, ASIP*, and *MC1R* by FISH to metaphase chromosomes in the alpaca, dromedary and Bactrian camel (Figure 4). For unambiguous chromosome identification, we used chromosome-specific reference markers from the alpaca cytogenetic map (Avila et al., 2014a). The *TYRP1* and *ASIP* genes were previously FISH mapped to alpaca chromosomes 4 and 19, respectively (Avila et al., 2014a). Here we mapped *TYRP1* to chr4 and *ASIP* to chr19 in both camel species (Figure 4A,B). The results are in agreement with karyotype conservation across Old and New World camelids (Bunch et al., 1985; Bianchi et al., 1986) and consistent with human-dromedary Zoo-FISH data on conserved synteny segments between these species (Balmus et al., 2007). The *MC1R* gene has not been chromosomally assigned in any camelid genome. Here we mapped *MC1R* to the very terminal region in chr21q in the alpaca, dromedary and Bactrian camel (Figure 4C) and revealed a hitherto unknown conserved synteny block between camelid chr21 and HSA16q.

## Discussion

Here we validated the recently published mutations for white and black/dark brown coat color in dromedaries (Almathen et al., 2018) using independent dromedary populations of US and Saudi Arabian origin. In addition, we designed for both mutations TaqMan^TM^ assays and confirmed their accuracy and efficiency for large cohort genotyping, suggesting that the high throughput, faster and cheaper TaqMan^TM^ assay should be the method of choice for any further genotyping of validated color-associated variants in large numbers of additional animals.

Overall, our results were consistent with the recently published data, but also refined and expanded it. Besides confirming the previously reported single variant in *MC1R* and two variants in *ASIP* (Almathen et al., 2018), we found six additional SNPs in *MC1R* non-coding regions, a new deletion in *ASIP* exon 4, and 5 novel variants in *TYRP1*.

The likely causative mutation for the white color in *MC1R* c.901C > T in dromedaries was, at the first sight intriguing because, according to published references (Rieder et al., 2001; Almathen et al., 2018), a mutation at the same position (c.901C > T) is responsible for recessive chestnut coat color in horses. This poses a question why the same missense mutation results in a depigmentation phenotype in the dromedary, but a pheomelanic phenotype in horses. However, closer inspection of the original publication for the horse chestnut mutation (Marklund et al., 1996; reviewed by Andersson, 2003) reveals that the horse chestnut and dromedary white mutations are different. The horse chestnut is due to p.Ser83Phe, which affects MC1R second transmembrane domain (Marklund et al., 1996), while the dromedary mutation p.Arg301Cys is in the last (seventh) transmembrane domain (Figure 2).

The dromedary mutation, however, shares functional and phenotypic similarity to recently reported *MC1R* sequence variants in Australian cattle dogs and Alaskan and Siberian huskies (Durig et al., 2018). Cream color in Australian cattle dogs is associated with a combination of c.916C > T (p.Arg306Ter) and a promoter variant affecting MITF binding site. White huskies, on the other hand, are homozygous for a deletion c.816-delCT. Even though causative sequence variants are different in Australian cattle dogs, huskies and dromedaries, they share essential similarities: all occur in the last transmembrane domain, negatively affect MC1R function and result in depigmentation phenotypes. The mutations in cream-colored Australian cattle dogs cause downregulation of *MC1R* transcription, while MC1R in white huskies has lost the last transmembrane domain and the cytoplasmic C-terminal tail (Durig et al., 2018). Though no functional data are available for white dromedaries, we theorize based on the predicted effect of p.Arg301Cys on the last transmembrane domain (Figure 2) and the resulting white phenotype, that the mutation is loss-of-function. Functional importance of this portion of the MC1R protein is illustrated by highly conserved sequence of 17 amino acids (p.296–312) across diverse mammalian species (Supplementary Figure S1). Notably, the dromedary differs from other mammals at p.301 because a white and not a wild-type animal was used for the reference sequence^11^.

In contrast to huskies where white color is a recessive trait (Durig et al., 2018), the dromedary white mutation is dominant because heterozygosity for the T-allele at c901C > T is sufficient for the white phenotype (Table 4 and Supplementary Table S1). Therefore, we suggest that the *MC1R* mutation in white dromedaries has dominant negative effect, i.e., it alters the function of the wild-type C-allele and has dominant or semi-dominant phenotype. Similar dominant negative effect on wild-type MC1R receptor cell surface expression or wild-type MC1R cAMP signaling has been described for several *MC1R* sequence variants in humans (Beaumont et al., 2007).

Another observation about dromedary *MC1R*, as also noted by Almathen et al. (2018), is the low level of sequence variation (just c901C > T) in the coding region, contrasting the 21 sequence variants found in the alpaca *MC1R* (Feeley and Munyard, 2009). However, a recent study of *MC1R* sequence variants across all four South American camelids (vicugna, guanaco, llama, and alpaca) suggests that variation in alpacas is the result of human selection for a variety of fiber colors, whereas in wild South American camelids (guanacos and free living vicugnas), there is a selection against non-synonymous substitutions in *MC1R* (Marin et al., 2018). Likewise, there is low sequence variation of *MC1R* in wild pigs, but many more variants in domestic pig breeds as a result of human selection (Andersson, 2003). Thus, we suggest that low sequence variation of *MC1R* in dromedaries is because human selection for coat color in this species is a more recent event in course of domestication.

On the other hand, sequence variants are present immediately outside the dromedary *MC1R* coding region, in 5′- and 3′-UTRs and in the promoter (Table 4). Whether any of these have regulatory roles in shaping pigmentation phenotypes, is a subject of future studies. This also applies to the 3′UTR variant g.538058G > A (Table 4), which showed association (*P* < 0.0004) with black coat color (Table 6). Though, it is also possible that the statistical significance may be influenced by relatedness between black animals.

The causative mutation for black coat color, as reported earlier (Almathen et al., 2018) and confirmed in this study (Table 7), is a frameshift deletion in *ASIP* exon 2, resulting in premature stop codon and truncated protein (Figure 3). Like in previous study (Almathen et al., 2018), we also observed a synonymous SNP 2 bp after the frameshift deletion (Table 4), but did not conduct association analysis because it was irrelevant for the premature stop codon. Similar, though not identical, loss-of-function mutations in *ASIP* underlie recessive black color in several domestic and wild species. For example, in alpacas (Feeley et al., 2011), sheep (Norris and Whan, 2008; Royo et al., 2008), Iranian Markhoz goats (Nazari-Ghadikolaei et al., 2018), donkeys (Abitbol et al., 2015), horses (Rieder et al., 2001), dogs (Kerns et al., 2004), cats (Eizirik et al., 2003), and impala antelope (Miller et al., 2016). Like in these species, we are confident that the black color in the dromedary is a recessive trait because the majority (34/38) of black dromedaries in this study were homozygous for the deletion (Table 7). However, 4 black animals in our study cohort did not follow this pattern (Table 7). Two of these carried another frameshift deletion in *ASIP* exon 4, resulting in abnormally long and likely non-functional ASIP protein (Figure 3). We suggest that the second frameshift deletion may be causative for black color in the absence of the first deletion, though it was not possible to conduct association analysis with just 2 individuals. Of the remaining two black dromedaries, one was heterozygous for the exon 2 deletion and the other had no mutations in *ASIP*. This is similar to observations in alpacas where homozygous recessive loss-of-function mutations in *ASIP* explain the majority but not all cases of the black phenotype (Feeley et al., 2011). Thus, like in alpacas, black coat color in dromedaries may be influenced by additional regulatory mutations and MC1R interactions with ASIP and α-melanocyte stimulating hormone (α-MSH). Nevertheless, at this point we did not conduct multi-locus testing because the majority of novel variants were non-coding, and because 12 animals in the discovery cohort would not give enough statistical power for these analyses. Besides, one should also consider possible errors in phenotyping.

We investigated the *TYRP1* gene as a possible contributor to various shades of brown coat color in the dromedary. The gene encodes for an important enzyme for the synthesis of eumelanin (del Marmol and Beermann, 1996) and *TYRP1* mutations are associated with brown or chocolate coat color on black genetic background in many mammals and other vertebrates (see Li et al., 2018). However, all *TYRP1* variants found in this study, were in non-coding regions (Table 4) and we did not detect the two SNPs in dromedary *TYRP1* exon1 as reported by a prior study (see Almathen et al., 2018). Nevertheless, both the non-coding SNPs and the exon 1 SNPs were not associated with any color phenotypes. Likewise, no candidate coat color mutations have been detected in alpaca *TYRP1* (Cransberg and Munyard, 2011). Despite these findings, *TYRP1* remains an important candidate gene for color phenotypes in camelids and should be included in future studies.

Finally, we comparatively FISH mapped the three coat color genes in three camelid species – the alpaca, the dromedary, and the Bactrian camel. In agreement with the known conservation of camelid karyotypes (Taylor et al., 1968; Bunch et al., 1985) and prior mapping of *TYRP1* and *ASIP* in alpacas (Avila et al., 2014a), the genes mapped to the same cytogenetic location in the same chromosomes in all species: *TYRP1* to chr4q21-q22, *ASIP* to chr19q12, and *MC1R* to chr21qter (Figure 4). While the locations of *TYRP1* and *ASIP* were in good agreement with human-dromedary Zoo-FISH (Balmus et al., 2007), mapping *MC1R* to chr21 came as a surprise. This is because camelid chr21 shares known conserved synteny with part of HSA1q only (Balmus et al., 2007; Avila et al., 2014a). Since human *MC1R* is located very terminal in the long arm of chr16 (HSA16q24.3; 89.9 Mb)^12^, we anticipated mapping *MC1R* to camelid chr9, which is homologous to HSA16q (Balmus et al., 2007; Avila et al., 2014a). Furthermore, camelid chr9 shares also homology with HSA19q, and HSA16q/HSA19q correspond to an ancestral eutherian synteny combination, which has been conserved in many eutherian karyotypes (Chowdhary et al., 1998; Ferguson-Smith and Trifonov, 2007). Our findings indicate that this ancestral synteny combination has undergone rearrangements during camelid karyotype evolution, so that a segment homologous to HSA16q containing *MC1R* has become a part of camelid chr21 and shares synteny with sequences corresponding to HSA1q. Inspection of the current dromedary genome assembly PRJNA234474_Ca_dromedarius_V1.0^13^ scaffolds confirmed FISH results for *MC1R* and showed that sequences corresponding to HSA1q: 145–147 Mb and HSA16q: 85–90 Mb are together in dromedary scaffold479 sequence NW_011591415.1^14^. Therefore, cytogenetic mapping of *MC1R* in camelids revealed a novel human-camelid synteny segment, confirmed sequence assembly of scaffold479, and anchored alpaca, dromedary and Bactrian camel scaffolds containing *TYRP1*, *ASIP*, and *MC1R* to chromosomes.

## Ethics Statement

Procurement of peripheral blood was performed according to the United States Government Principles for the Utilization and Care of Vertebrate Animals Used in Testing, Research and Training. All procedures were approved by Institutional Animal Care and Use Committee as AUP#2011-96, AUP#2018-0342CA and CRRC#09-47 at Texas A&M University.

## Author Contributions

TR and FA initiated and designed the study. FA, CC, RJ, AH, MM, GG, and FPdL conducted the experimental work and data analysis. TR, FA, and CC wrote the manuscript with input from all authors.

## Conflict of Interest Statement

The authors declare that the research was conducted in the absence of any commercial or financial relationships that could be construed as a potential conflict of interest. The handling Editor and reviewer PO-tW declared their involvement as co-editors in the Research Topic, and confirm the absence of any other collaboration.

## Footnotes

1 http://www.espcr.org/micemut/

2 https://www.ncbi.nlm.nih.gov/genome

3 http://www.ncbi.nlm.nih.gov

4 http://genome.ucsc.edu/

5 http://www.ensembl.org/index.html

6 http://provean.jcvi.org/index.php

7 https://www.ncbi.nlm.nih.gov/

8 http://www.ensembl.org/index.html

9 http://www.cbs.dtu.dk/services/TMHMM/TMHMM2.0b.guide.php

10 http://bacpacresources.org/library.php?id=448

11 https://www.ncbi.nlm.nih.gov/genome/10740?genome_assembly_id=212034

12 https://genome.ucsc.edu/

13 https://www.ncbi.nlm.nih.gov/assembly/GCF_000767585.1/

14 https://www.ncbi.nlm.nih.gov/nuccore/NW_011591415.1?report=graph

## Supplementary Material

Figure S1 *MC1R* comparative. Comparative alignment of *MC1R* transmembrane domain 7 amino acid sequences in diverse mammalian and vertebrate species. The p.301 position is indicated by a vertical arrow and highlighted; horizontal arrow shows the p.301R > C mutation in the dromedary. Note that Arginine is highly conserved across species, except in humans who have Histidine, which is another positively charged amino acid.

Figure S2 Contingency analysis output files in JMP program for the sequence variants analyzed in this study. Each output file comprises a Mosaic Plot, a Contingency Table, and Statistics Tests; N, the total number of observations; DF, records the degrees of freedom associated with the test; -LogLike, negative log-likelihood; Rsquare (U), shows portion of the total uncertainty attributed to the model fit. ChiSquare, two chi-square statistical tests; Prob > ChiSq, lists the probability of obtaining, by chance alone, a Chi-square value greater than the one computed if no relationship exists between phenotype and genotype.

TABLE S1 Composite information about the dromedaries used in this study (color, ID, country of origin, sex, photo, **MC1R** g901C > T genotype; **ASIP** exon 2 g.174495T > Del nonsense mutation, and **MC1R** 3′UTRG > A genotype); ^∗^ denote the animals for which genotypes were determined both by Sanger sequencing and TaqMan^TM^ assay.

TABLE S2 Expected and observed allele and genotype frequencies for all variants found in this study.
