# Supplementary figures and images for: Comparative FISH-Mapping of MC1R, ASIP, and TYRP1 in New and Old World Camelids and Association Analysis With Coat Color Phenotypes in the Dromedary (Camelus dromedarius)
Source: Front Genet. 2019 Apr 16;10:340. doi: 10.3389/fgene.2019.00340 (PMC6477024; doi:10.3389/fgene.2019.00340)

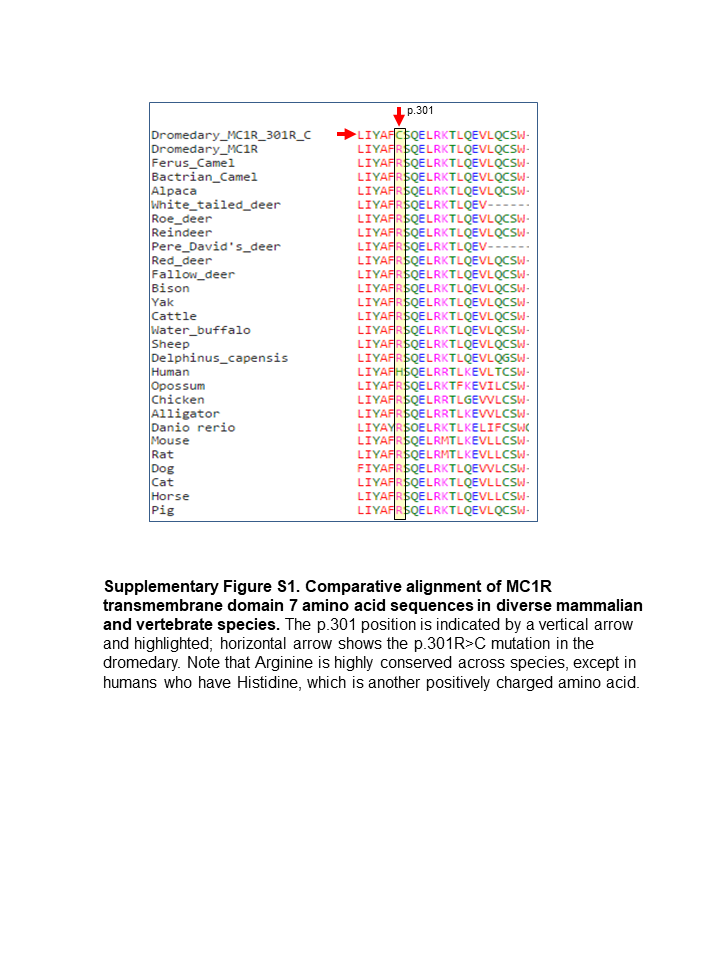

Supplement: Figure S1 — MC1R comparative. Comparative alignment of MC1R transmembrane domain 7 amino acid sequences in diverse mammalian and vertebrate species. The p.301 position is indicated by a vertical arrow and highlighted; horizontal arrow shows the p.301R > C mutation in the dromedary. Note that Arginine is highly conserved across species, except in humans who have Histidine, which is another positively charged amino acid. [file Image_1.TIF]
