# Supplementary material for: Comparative FISH-Mapping of MC1R, ASIP, and TYRP1 in New and Old World Camelids and Association Analysis With Coat Color Phenotypes in the Dromedary (Camelus dromedarius)
Source: Front Genet. 2019 Apr 16;10:340. doi: 10.3389/fgene.2019.00340 (PMC6477024; doi:10.3389/fgene.2019.00340)
Supplement: Figure S2 — Contingency analysis output files in JMP program for the sequence variants analyzed in this study. Each output file comprises a Mosaic Plot, a Contingency Table, and Statistics Tests; N, the total number of observations; DF, records the degrees of freedom associated with the test; -LogLike, negative log-likelihood; Rsquare (U), shows portion of the total uncertainty attributed to the model fit. ChiSquare, two chi-square statistical tests; Prob > ChiSq, lists the probability of obtaining, by chance alone, a Chi-square value greater than the one computed if no relationship exists between phenotype and genotype. [file Data_Sheet_1.PDF]

Supplementary Figure S2: Contingency analysis output files in JMP program for the sequence variants analyzed in this study. Each output file comprises a **Mosaic Plot**, a **Contingency Table**, and **Statistics Tests**; **N -** the total number of observations; **DF -** records the degrees of freedom associated with the test; **-LogLike -** negative log-likelihood; **Rsquare (U) -** shows portion of the total uncertainty attributed to the model fit. **ChiSquare** – two chi-square statistical tests; **Prob>ChiSq -** lists the probability of obtaining, by chance alone, a Chi-square value greater than the one computed if no relationship exists between phenotype and genotype.

MC1R Promotor

g.535236C>T

Contingency Analysis of Population Colors By Genotypes

Mosaic Plot

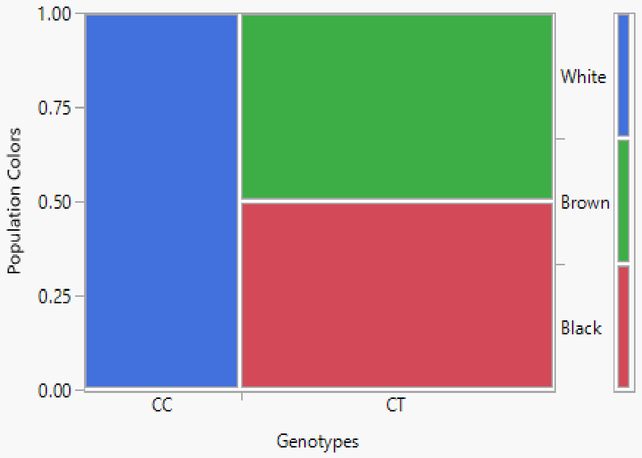

Contingency Table

Population Colors

Genotypes	Count<br>Total %<br>Col %<br>Row %	Black	Brown	White	Total
	CC	0<br>0.00<br>0.00<br>0.00	0<br>0.00<br>0.00<br>0.00	4<br>33.33<br>100.00<br>100.00	4<br>33.33
	CT	4<br>33.33<br>100.00<br>50.00	4<br>33.33<br>100.00<br>50.00	0<br>0.00<br>0.00<br>0.00	8<br>66.67
	Total	4<br>33.33	4<br>33.33	4<br>33.33	12

Tests

N	DF	-LogLike	RSquare (U)
12	2	7.6381700	0.5794

Test	ChiSquare	Prob>ChiSq
Likelihood Ratio	15.276	0.0005*
Pearson	12.000	0.0025*

Warning: 20% of cells have expected count less than 5, ChiSquare suspect.
Warning: Average cell count less than 5, LR ChiSquare suspect.

MC1R Promotor

g.536482G>A

Contingency Analysis of Population Colors By Genotypes

Mosaic Plot

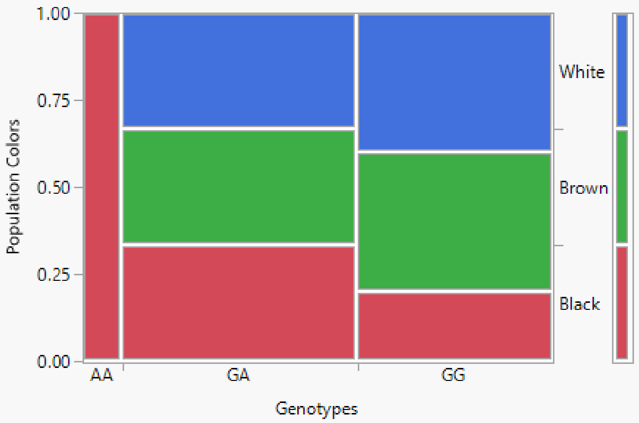

Contingency Table

Population Colors

Genotypes	Count<br>Total %<br>Col %<br>Row %	Black	Brown	White	Total
	AA	1<br>8.33<br>25.00<br>100.00	0<br>0.00<br>0.00<br>0.00	0<br>0.00<br>0.00<br>0.00	1<br>8.33
	GA	2<br>16.67<br>50.00<br>33.33	2<br>16.67<br>50.00<br>33.33	2<br>16.67<br>50.00<br>33.33	6<br>50.00
	GG	1<br>8.33<br>25.00<br>20.00	2<br>16.67<br>50.00<br>40.00	2<br>16.67<br>50.00<br>40.00	5<br>41.67
	Total	4<br>33.33	4<br>33.33	4<br>33.33	12

Tests

N	DF	-LogLike	RSquare (U)
12	4	1.3170729	0.0999

Test	ChiSquare	Prob>ChiSq
Likelihood Ratio	2.634	0.6208
Pearson	2.400	0.6626

Warning: 20% of cells have expected count less than 5, ChiSquare suspect.
Warning: Average cell count less than 5, LR ChiSquare suspect.

MC1R Promotor & 5' UTR

g.536623G>A; g.537027G>A; g.537028A>T
3 Variants have identical P-values

Contingency Analysis of Population Colors By Genotypes

Mosaic Plot

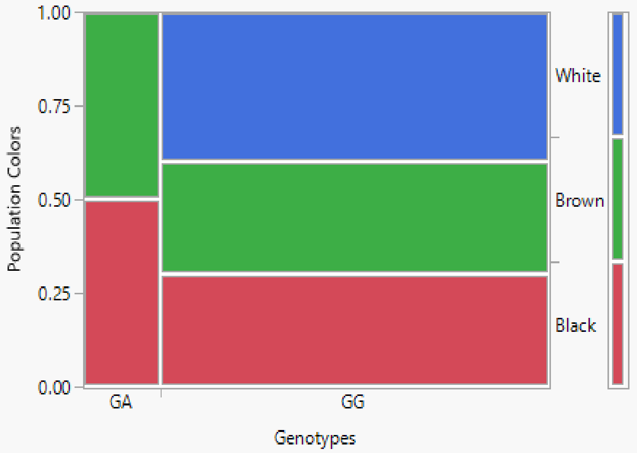

Contingency Table

Population Colors

Genotypes	Count<br>Total %<br>Col %<br>Row %	Black	Brown	White	Total
	GA	1<br>8.33<br>25.00<br>50.00	1<br>8.33<br>25.00<br>50.00	0<br>0.00<br>0.00<br>0.00	2<br>16.67
	GG	3<br>25.00<br>75.00<br>30.00	3<br>25.00<br>75.00<br>30.00	4<br>33.33<br>100.00<br>40.00	10<br>83.33
	Total	4<br>33.33	4<br>33.33	4<br>33.33	12

Tests

N	DF	-LogLike	RSquare (U)
12	2	0.90805335	0.0689

Test	ChiSquare	Prob>ChiSq
Likelihood Ratio	1.816	0.4033
Pearson	1.200	0.5488

Warning: 20% of cells have expected count less than 5, ChiSquare suspect.
Warning: Average cell count less than 5, LR ChiSquare suspect.

MC1R Exon

c.901C>T

Contingency Analysis of Population Colors By Genotypes

Mosaic Plot

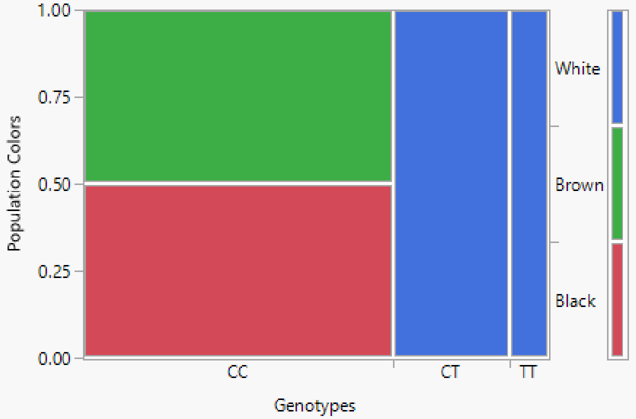

Contingency Table

Population Colors

Genotypes	Count / Total % / Col % / Row %	Black	Brown	White	Total
CC	Count	4	4	0	8
	Total %	33.33	33.33	0.00	66.67
	Col %	100.00	100.00	0.00	
	Row %	50.00	50.00	0.00	
CT	Count	0	0	3	3
	Total %	0.00	0.00	25.00	25.00
	Col %	0.00	0.00	75.00	
	Row %	0.00	0.00	100.00	
TT	Count	0	0	1	1
	Total %	0.00	0.00	8.33	8.33
	Col %	0.00	0.00	25.00	
	Row %	0.00	0.00	100.00	
Total	Count	4	4	4	12
	Total %	33.33	33.33	33.33	

Tests

N	DF	-LogLike	RSquare (U)
12	4	7.6381700	0.5794

Test	ChiSquare	Prob>ChiSq
Likelihood Ratio	15.276	0.0042*
Pearson	12.000	0.0174*

Warning: 20% of cells have expected count less than 5, ChiSquare suspect.
Warning: Average cell count less than 5, LR ChiSquare suspect.

MC1R 3' UTR

g.538058G>A

Contingency Analysis of Population Colors By Genotypes

Mosaic Plot

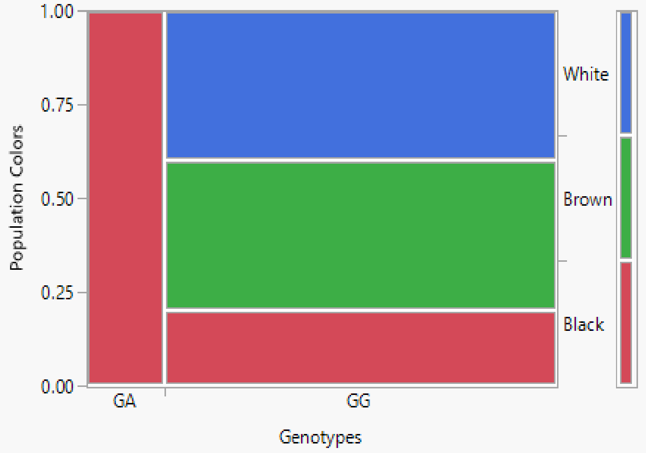

Contingency Table

Population Colors

Genotypes	Count / Total % / Col % / Row %	Black	Brown	White	Total
GA	Count	2	0	0	2
	Total %	16.67	0.00	0.00	16.67
	Col %	50.00	0.00	0.00	
	Row %	100.00	0.00	0.00	
GG	Count	2	4	4	10
	Total %	16.67	33.33	33.33	83.33
	Col %	50.00	100.00	100.00	
	Row %	20.00	40.00	40.00	
Total	Count	4	4	4	12
	Total %	33.33	33.33	33.33	

Tests

N	DF	-LogLike	RSquare (U)
12	2	2.6341458	0.1998

Test	ChiSquare	Prob>ChiSq
Likelihood Ratio	5.268	0.0718
Pearson	4.800	0.0907

Warning: 20% of cells have expected count less than 5, ChiSquare suspect.
Warning: Average cell count less than 5, LR ChiSquare suspect.

ASIP Exon 2

c.23T_del; c.25A>G

*2 Variants have identical P-values

Contingency Analysis of Population Colors By Genotypes

Mosaic Plot

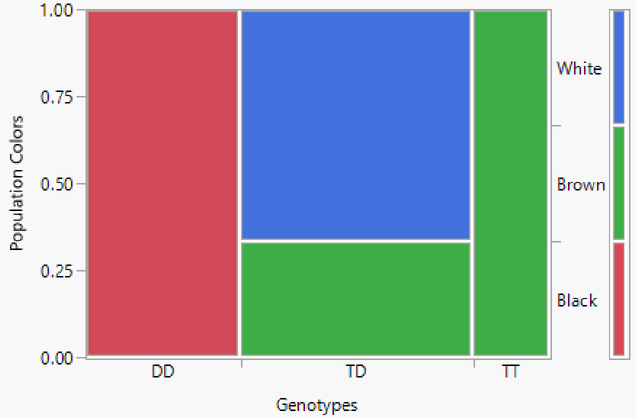

Contingency Table

Population Colors

Genotypes	Count / Total % / Col % / Row %	Black	Brown	White	Total
DD	Count	4	0	0	4
	Total %	33.33	0.00	0.00	33.33
	Col %	100.00	0.00	0.00	
	Row %	100.00	0.00	0.00	
TD	Count	0	2	4	6
	Total %	0.00	16.67	33.33	50.00
	Col %	0.00	50.00	100.00	
	Row %	0.00	33.33	66.67	
TT	Count	0	2	0	2
	Total %	0.00	16.67	0.00	16.67
	Col %	0.00	50.00	0.00	
	Row %	0.00	100.00	0.00	
Total	Count	4	4	4	12
	Total %	33.33	33.33	33.33	

Tests

N	DF	-LogLike	RSquare (U)
12	4	9.3642625	0.7103

Test	ChiSquare	Prob>ChiSq
Likelihood Ratio	18.729	0.0009*
Pearson	16.000	0.0030*

Warning: 20% of cells have expected count less than 5, ChiSquare suspect.
Warning: Average cell count less than 5, LR ChiSquare suspect.

ASIP Exon 4

g.178388C_del

Contingency Analysis of Population Colors By Genotypes

Mosaic Plot

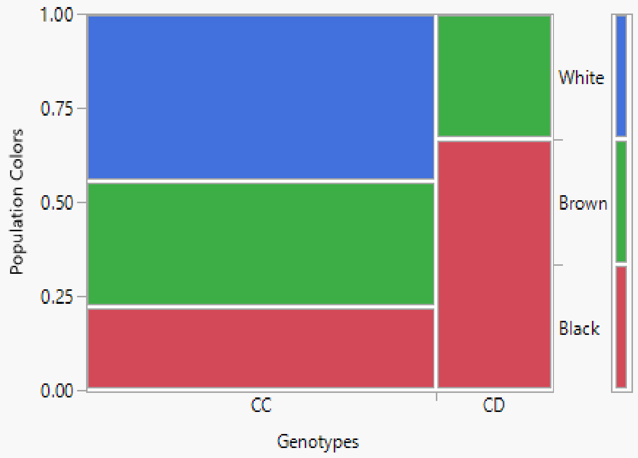

Contingency Table

Genotypes	Count / Total % / Col % / Row %	Black	Brown	White	Total
CC	Count	2	3	4	9
	Total %	16.67	25.00	33.33	75.00
	Col %	50.00	75.00	100.00	
	Row %	22.22	33.33	44.44	
CD	Count	2	1	0	3
	Total %	16.67	8.33	0.00	25.00
	Col %	50.00	25.00	0.00	
	Row %	66.67	33.33	0.00	
Total	Count	4	4	4	12
	Total %	33.33	33.33	33.33	

Tests

N	DF	-LogLike	RSquare (U)
12	2	1.7260924	0.1309

Test	ChiSquare	Prob>ChiSq
Likelihood Ratio	3.452	0.1780
Pearson	2.667	0.2636

Warning: 20% of cells have expected count less than 5, ChiSquare suspect.
Warning: Average cell count less than 5, LR ChiSquare suspect.

TYRP1 Intron 1

g.6544484G>A

Contingency Analysis of Population Colors By Genotypes

Mosaic Plot

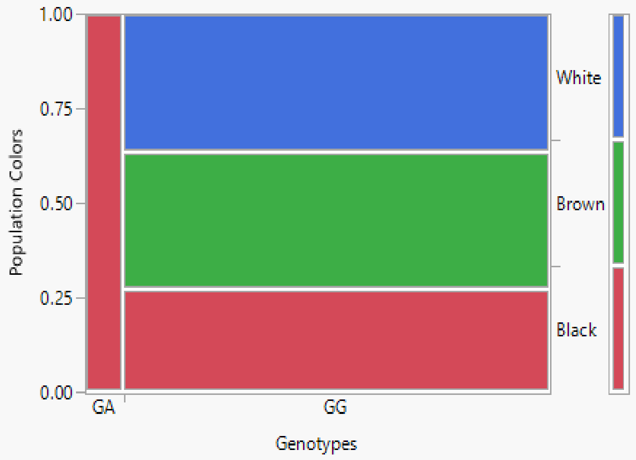

Contingency Table

Genotypes	Count / Total % / Col % / Row %	Black	Brown	White	Total
GA	Count	1	0	0	1
	Total %	8.33	0.00	0.00	8.33
	Col %	25.00	0.00	0.00	
	Row %	100.00	0.00	0.00	
GG	Count	3	4	4	11
	Total %	25.00	33.33	33.33	91.67
	Col %	75.00	100.00	100.00	
	Row %	27.27	36.36	36.36	
Total	Count	4	4	4	12
	Total %	33.33	33.33	33.33	

Tests

N	DF	-LogLike	RSquare (U)
12	2	1.1926912	0.0905

Test	ChiSquare	Prob>ChiSq
Likelihood Ratio	2.385	0.3034
Pearson	2.182	0.3359

Warning: 20% of cells have expected count less than 5, ChiSquare suspect.
Warning: Average cell count less than 5, LR ChiSquare suspect.

TYRP1 Intron 2

g.6544049T>C

Contingency Analysis of Population Colors By Genotypes

Mosaic Plot

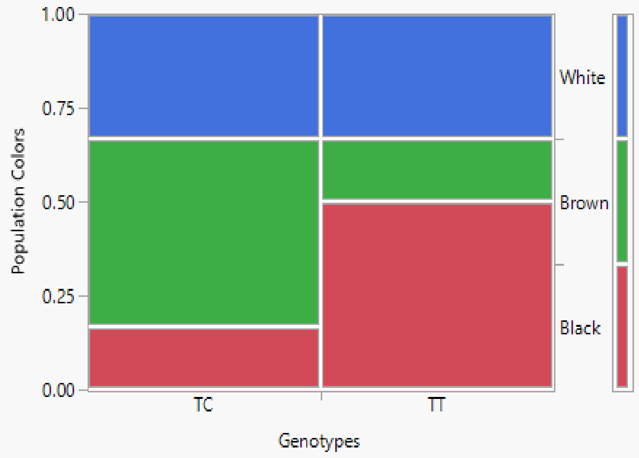

Contingency Table

Genotypes	Count / Total % / Col % / Row %	Black	Brown	White	Total
TC	Count	1	3	2	6
	Total %	8.33	25.00	16.67	50.00
	Col %	25.00	75.00	50.00	
	Row %	16.67	50.00	33.33	
TT	Count	3	1	2	6
	Total %	25.00	8.33	16.67	50.00
	Col %	75.00	25.00	50.00	
	Row %	50.00	16.67	33.33	
Total	Count	4	4	4	12
	Total %	33.33	33.33	33.33	

Tests

N	DF	-LogLike	RSquare (U)
12	2	1.0464963	0.0794

Test	ChiSquare	Prob>ChiSq
Likelihood Ratio	2.093	0.3512
Pearson	2.000	0.3679

Warning: 20% of cells have expected count less than 5, ChiSquare suspect.
Warning: Average cell count less than 5, LR ChiSquare suspect.

TYRP1 Intron 3

g.65372260_g.65372261insCA

Contingency Analysis of Population Colors By Genotypes

Mosaic Plot

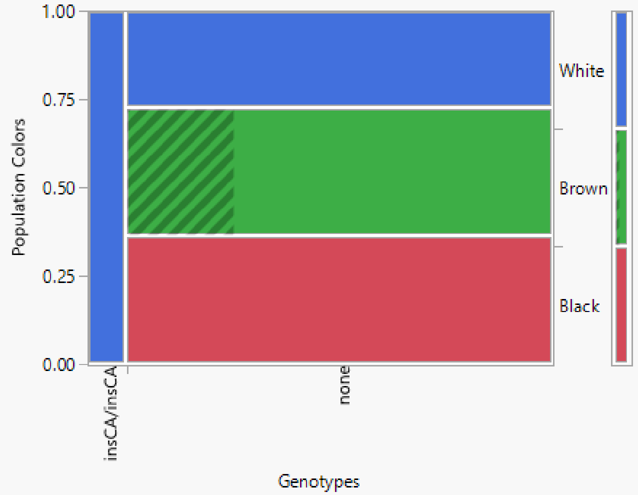

Contingency Table

Population Colors

Genotypes	Count / Total % / Col % / Row %	Black	Brown	White	Total
insCA/insCA	Count	0	0	1	1
	Total %	0.00	0.00	8.33	8.33
	Col %	0.00	0.00	25.00	
	Row %	0.00	0.00	100.00	
none	Count	4	4	3	11
	Total %	33.33	33.33	25.00	91.67
	Col %	100.00	100.00	75.00	
	Row %	36.36	36.36	27.27	
Total	Count	4	4	4	12
	Total %	33.33	33.33	33.33	

Tests

N	DF	-LogLike	RSquare (U)
12	2	1.1926912	0.0905

Test	ChiSquare	Prob>ChiSq
Likelihood Ratio	2.385	0.3034
Pearson	2.182	0.3359

Warning: 20% of cells have expected count less than 5, ChiSquare suspect.
Warning: Average cell count less than 5, LR ChiSquare suspect.

TYRP1 Intron 4

g.6537183T>C

Contingency Analysis of Population Colors By Genotypes

Mosaic Plot

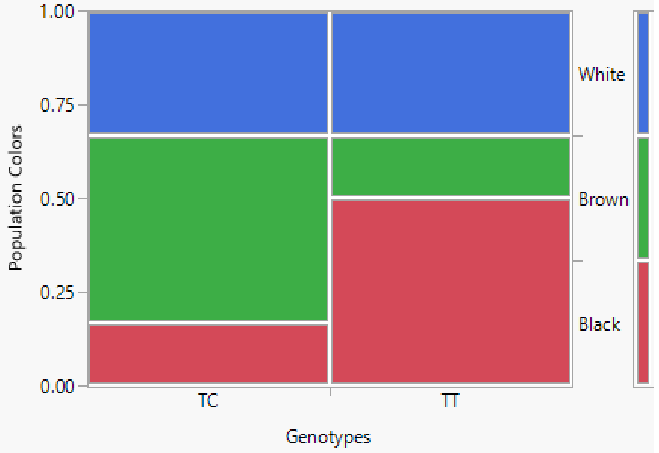

Contingency Table

Population Colors

Genotypes	Count / Total % / Col % / Row %	Black	Brown	White	Total
TC	Count	1	3	2	6
	Total %	8.33	25.00	16.67	50.00
	Col %	25.00	75.00	50.00	
	Row %	16.67	50.00	33.33	
TT	Count	3	1	2	6
	Total %	25.00	8.33	16.67	50.00
	Col %	75.00	25.00	50.00	
	Row %	50.00	16.67	33.33	
Total	Count	4	4	4	12
	Total %	33.33	33.33	33.33	

Tests

N	DF	-LogLike	RSquare (U)
12	2	1.0464963	0.0794

Test	ChiSquare	Prob>ChiSq
Likelihood Ratio	2.093	0.3512
Pearson	2.000	0.3679

Warning: 20% of cells have expected count less than 5, ChiSquare suspect.
Warning: Average cell count less than 5, LR ChiSquare suspect.

TYRP1 3'UTR

g.6529345A>T

Contingency Analysis of Population Colors By Genotypes

Mosaic Plot

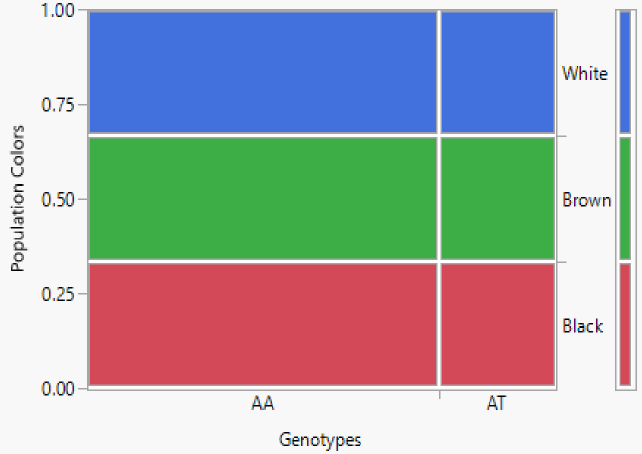

Contingency Table

Population Colors

Genotypes	Count / Total % / Col % / Row %	Black	Brown	White	Total
AA	Count	3	3	3	9
	Total %	25.00	25.00	25.00	75.00
	Col %	75.00	75.00	75.00	
	Row %	33.33	33.33	33.33	
AT	Count	1	1	1	3
	Total %	8.33	8.33	8.33	25.00
	Col %	25.00	25.00	25.00	
	Row %	33.33	33.33	33.33	
Total	Count	4	4	4	12
	Total %	33.33	33.33	33.33	

Tests

N	DF	-LogLike	RSquare (U)
12	2	1.7764e-15	0.0000

Test	ChiSquare	Prob>ChiSq
Likelihood Ratio	0.000	1.0000
Pearson	0.000	1.0000

Warning: 20% of cells have expected count less than 5, ChiSquare suspect.
Warning: Average cell count less than 5, LR ChiSquare suspect.
